# The impact of the cryopreservation period on the CD34+ cell viability of cryopreserved cord blood units

**DOI:** 10.1111/vox.70281

**Published:** 2026-05-08

**Authors:** Hye Ryun Lee, Eun Youn Roh, Namhee Kim, Hyunwoong Park, Jong Hyun Yoon, Sue Shin

**Affiliations:** ^1^ Department of Laboratory Medicine National Medical Center Seoul Republic of Korea; ^2^ Department of Laboratory Medicine Seoul National University College of Medicine Seoul Republic of Korea; ^3^ Department of Laboratory Medicine SMG‐SNU Boramae Medical Center Seoul Republic of Korea; ^4^ Seoul Metropolitan Government Public Cord Blood Bank (Allcord) Seoul Republic of Korea

**Keywords:** CD34+ cells, cord blood (CB), cryopreservation, cryopreservation period, quality, viability

## Abstract

**Background and Objectives:**

Cord blood (CB) is an important source for haematopoietic stem cell transplantation because of its long‐term cryopreservation. However, concerns remain regarding the potential decline in CB quality with prolonged cryopreservation. This study aimed to evaluate the post‐thaw viability of CD45+ and CD34+ cells in CB units and to investigate how their viabilities change over prolonged cryopreservation.

**Materials and Methods:**

We enrolled 726 CB units that underwent pre‐transplantation examination from May 2006 through June 2023. Post‐thaw viabilities of CD45+ and CD34+ cells were analysed in relation to cryopreservation period and across 4‐year cryopreservation groups.

**Results:**

The mean post‐thaw viabilities were 60.5% for CD45+ cells and 91.2% for CD34+ cells. CD45+ cell viability showed a positive correlation with the cryopreservation period (*r* = 0.304, *p* < 0.001); however, no consistent trend was observed among the cryopreservation period groups. In contrast, CD34+ cell viability showed a significant negative correlation with the cryopreservation period (*r* = − 0.306, *p* <0.001) and decreased, with units cryopreserved for 1–4 years and 5–8 years having significantly higher viabilities than those cryopreserved for 9–12 years and 13–16 years.

**Conclusion:**

Our findings demonstrate that the post‐thaw viabilities of CD45+ and CD34+ cells in cryopreserved CB units exceed internationally accepted standards, with CD34+ cell viability showing a tendency to decline with prolonged cryopreservation. These results underscore the importance of securing an adequate CD34+ cell dose at the time of cryopreservation to maintain the long‐term quality and clinical utility of CB units.

**Highlights**

- Cord blood (CB) units cryopreserved for up to 16 years maintain high post‐thaw viabilities of CD45+ and CD34+ cells, exceeding internationally recognized quality standards for transplantation.
- While CD45+ cell viability did not exhibit a consistent decreasing or increasing trend among the cryopreservation period groups, CD34+ cell viability decreased, with units cryopreserved for 1–4 years and 5–8 years having significantly higher viabilities than those cryopreserved for 9–12 years and 13–16 years.
- It is important to secure an adequate CD34+ cell dose at the time of cryopreservation to maintain the long‐term quality and clinical utility of CB units.

## INTRODUCTION

Cord blood (CB) has become a valuable source of haematopoietic stem cell transplantation (HSCT), offering advantages such as relatively easy procurement due to cryopreservation for prolonged periods. However, the impact of long‐term cryopreservation on the quality of CB has been of critical concern, as it may affect the functional activity of HSCs.

To ensure the quality of cryopreserved CB units, CB banks perform the pre‐transplantation examination using contiguous segment or representative sample prior to releasing them for HSCT. The number of total nucleated cells (TNCs) and CD34+ cells, cell viability, the number of colony‐forming units (CFUs) and other analyses have been used as quality markers of cryopreserved CB units. While the number of TNCs and/or CD34+ cells are commonly used to assess HSC activity in haematopoietic products, these numbers may not represent the functional activity of HSCs [1, 2]. High cell viability is crucial for maintaining the potency of CB units as a source of HSCs, impacting the engraftment and prognosis after HSCT [3, 4]. The international standards of NetCord and Foundation for the Accreditation of Cellular Therapy (FACT) specify the viability of TNCs as one of the quality requirements for CB after processing before cryopreservation, and the viability of CD34+ cells as one of the specification requirements for the quality of CB after thawing before release for transplantation [5]. Paul Ehrlich Institute (PEI) of Germany has been specifying the viability of TNCs and the viability of CD34+ cells as the requirements for the quality of CB after processing before cryopreservation and after thawing before release for transplantation. Additionally, the viability of CD45+ cells, which represent the leukocyte population gating through flow cytometry analysis, has been specified as one of the requirements for the quality of CB after thawing before release for transplantation [6].

In this study, we assessed the viability of CD45+ and CD34+ cells of thawing CB units and investigated the change in the viability of CD45+ and CD34+ cells over the cryopreservation period to evaluate the impact of long‐term cryopreservation.

## MATERIALS AND METHODS

### Subject and study design

The Seoul Metropolitan Government Public Cord Blood Bank (Allcord), established in May 2006, operates the largest public CB bank in South Korea. Prior to the release of cryopreserved CB to transplantation centres, Allcord routinely assesses the quality of selected CB units by measuring TNC and CD34+ cell counts, TNC and CD34+ cell recovery rates, as well as TNC viability using a contiguous segment of each unit.

For the present study, we additionally evaluated the viability of CD45+ and CD34+ cells in thawed CB units as part of the quality assessment. Among 999 CB units that underwent pre‐transplantation examination for HSCT through June 2023, we analysed 726 units based on data availability and completeness. These CB units had been donated to Allcord between May 2006 and December 2017.

We analysed these quality markers based on the cryopreservation period, defined as the time between cryopreservation and pre‐transplantation testing. The cryopreservation periods were categorized into four groups in 4‐year intervals: 1–4 years, 5–8 years, 9–12 years and 13–16 years. Differences in CD45+ and CD34+ cell viability among these groups were compared.

This study was exempted from review by the Institutional Review Board at Seoul Metropolitan Government Seoul National University (SMG‐SNU) Boramae Medical Center (IRB no. 20‐2023‐35).

### CB collection, processing and testing before cryopreservation

CB was collected in utero in the delivery room. After volume reduction of the collected CB by depleting both red blood cells and plasma, tests that included evaluating the TNC count, the CD34+ cell count and TNC viability were performed. The TNC (neutrophils, lymphocytes and monocytes) count was carried out using an automated cell counter (XE‐2100; Sysmex, Kobe, Japan). The CD34+ cell count was done by flowcytometry (FC‐500, Beckman Coulter, Fullerton, CA, USA) using the surface antibodies anti‐CD45 fluorescein isothiocyanate (FITC) (BD Biosciences, San Jose, CA) and anti‐CD34 phycoerythrin (PE) (BD Biosciences), based on the International Society of Hematotherapy and Graft Engineering (ISHAGE) method. Non‐viable cells were excluded by incorporating the viability dye 7‐aminoactinomycin D (7‐AAD) (BD Biosciences) in the flow cytometry procedure. TNC viability was tested by 0.4% trypan blue staining. The procedures were duplicated by two independent technicians. Units were frozen using a controlled‐rate freezer (CryoMed, Thermo Forma, Marietta, OH). After being frozen, the CB units were stored in the liquid phase of liquid nitrogen. The time between collection and end of freezing of CB units did not exceed 36 h. Data on TNC counts, CD34+ cell counts and TNC viability before cryopreservation were retrieved from the Allcord database.

### Pre‐transplantation examination of selected CB units

A contiguous segment of each selected CB unit was thawed in a 37°C water bath. TNC counts, CD34+ cell counts and TNC viability were measured after thawing for quality testing of CB units prior to release for transplantation, using the same method as those applied before cryopreservation. Data on TNC counts, CD34+ cell counts and TNC viability after thawing were retrieved from the Allcord database.

### Additional analysis for the viability of CD45+ and CD34+ cells of CB units after thawing

Cell viability is usually determined by defining the living cells through dye exclusion. Non‐viable cells are excluded by incorporating the viability dye trypan blue under microscopic examination and by incorporating the viability dye 7‐AAD in the flow cytometry procedure for CD34+ enumeration [5]. The *Act on cord blood management and research* established in July 2011 specifies in the appendix 1 for CB management in Korea that TNC viability is the sole quality marker regarding cell viability in CB [7]. In addition to TNC viability, CB banks in Korea are required to perform quality testing after processing before cryopreservation, including TNC and CD34+ cell counts. However, quality testing to determine the suitability of CB units for transplantation is conducted solely at the discretion of each CB bank, as there are no specified requirements for such testing. Allcord has performed pre‐transplantation examination, including TNC counts, TNC viability and CD34+ cell counts, as part of quality assurance as mentioned in the previous section.

**FIGURE 1:**
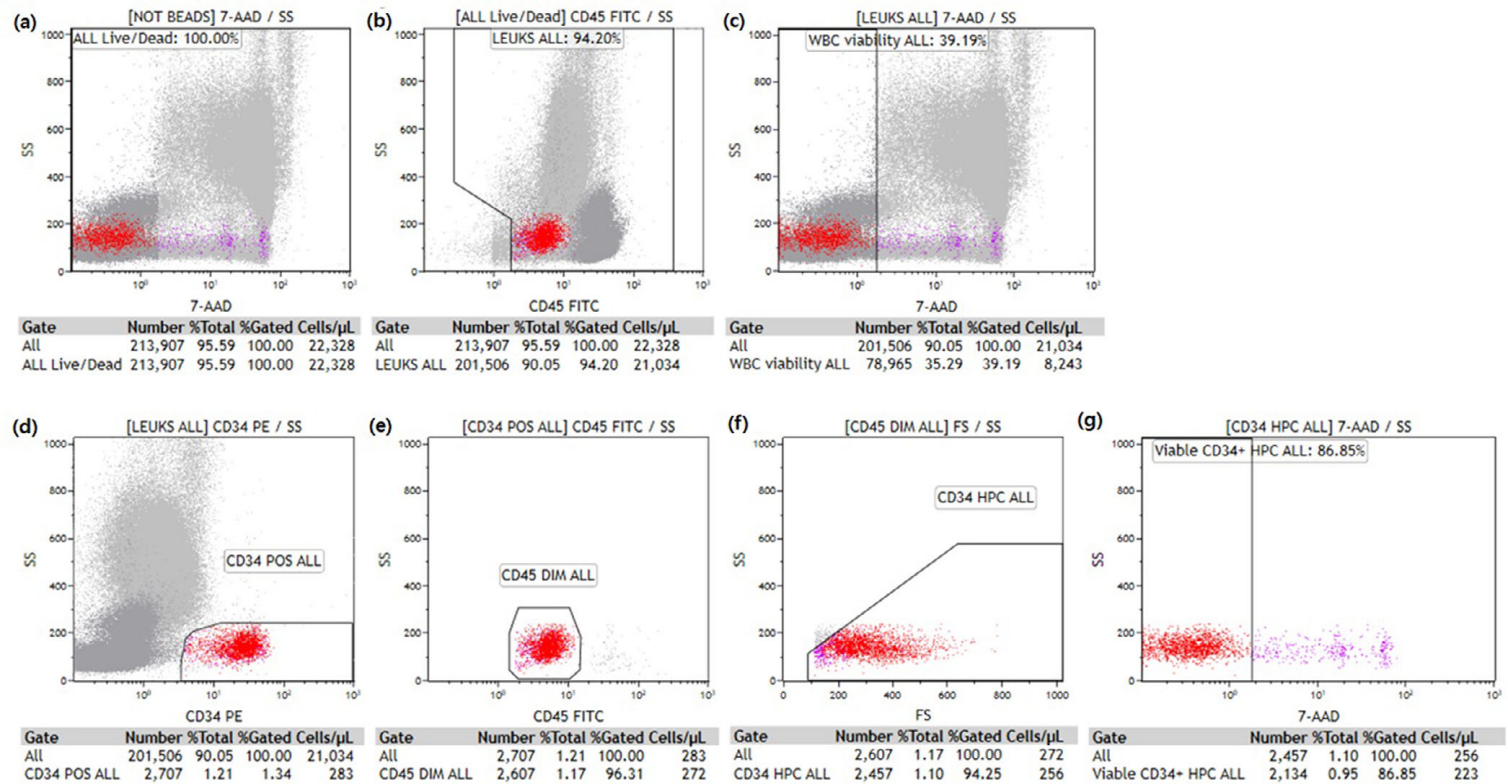
Example analysis of CD45+ and CD34+ cell viabilities using linear mode data files from flow cytometry in pre‐transplantation examination. (a) 7‐aminoactinomycin D (7‐AAD) versus side scatter (SS) dot plot showing all cellular events after exclusion of counting beads (‘NOT BEADS’). All events fall within the live/dead discrimination gate (‘ALL Live/Dead’), corresponding to 100% live/dead–gated events in this sample, as defined by the use of 7‐AAD dye to separate viable from non‐viable cells. (b) CD45 versus SS dot plot of all live/dead–gated events (‘ALL Live/Dead’). A broad gate (‘LEUKS ALL’) is drawn around CD45+, low‐to‐intermediate SS leukocytes, which are used as the denominator population for CD34+ cell enumeration in accordance with the International Society of Hematotherapy and Graft Engineering (ISHAGE) protocol. (c) 7‐AAD versus SS dot plot of the leukocyte gate (‘LEUKS ALL’). Within this CD45+ cell population, viable CD45+ cells are defined as 7‐AAD‐negative events (gate ‘WBC viability ALL’), yielding a CD45+ cell viability of 39.19%. (d) CD34 versus SS dot plot showing all leukocyte events (‘LEUKS ALL’). A gate is set around low‐SS, CD34+ events (‘CD34 POS ALL’), which represent putative CD34+ cells while excluding debris and non‐leukocytic events in accordance with the ISHAGE recommendations. (e) CD45 versus SS dot plot of the CD34‐gated population (‘CD34 POS ALL’). Within these CD34+ events, haematopoietic progenitor cells are identified as CD45 dim, low‐SS cells (‘CD45 DIM ALL’). (f) Forward scatter (FS) versus SS dot plot of the CD45 dim population (‘CD45 DIM ALL’). A region is drawn around cells with low to intermediate FS and low SS (‘CD34 hematopoietic progenitor cell [HPC] ALL’), corresponding to small‐to‐medium‐sized cells with scatter properties slightly higher than lymphocytes, as defined by the ISHAGE gating strategy. (g) 7‐AAD versus SS dot plot of the CD34 HPC gate (‘CD34 HPC ALL’). Viable CD34+ cells are defined as 7‐AAD‐negative events within the CD34 HPC region (‘Viable CD34+ HPC ALL’), allowing exclusion of non‐viable cells and yielding a CD34+ cell viability of 86.85%.

In this study, we additionally analysed the lineage‐specific viability of CD45+ and CD34+ cells using Kaluza analysis software (Beckman Coulter, Fullerton, CA, USA) with linear mode data (LMD) files from the flowcytometry. These data had been previously acquired for CD34+ cell count after thawing as part of the pre‐transplantation examination. CD45+ cells were analysed using anti‐CD45 FITC and side scatter (SS) dot plot. After gating the CD45+ cells, we analysed viable CD45+ cells on the 7‐AAD versus SS dot plot. Following the determination of CD34+ cells according to the ISHAGE method, viable CD34+ cells were evaluated on the 7‐AAD versus SS dot plot (Figure 1).

### Statistical analysis

A correlation analysis was conducted to examine the relationship between the cryopreservation period and the recovery rates of TNC, CD34+ cell count and the difference in TNC viability before cryopreservation and after thawing of the CB units. Additionally, we investigated the correlation between the cryopreservation period and the viability of CD45+ and CD34+ cells. One‐way analysis of variance (ANOVA) and multiple comparison analyses were performed to investigate differences in the viability of CD45+ and CD34+ cells according to the cryopreservation period groups. Differences were considered to be statistically significant when the *p*‐value was <0.05. All statistical calculations were performed using the Statistical Package for Social Sciences (SPSS) Statistics (version 27; IBM Corp., Chicago, IL, USA).

## RESULTS

### Characteristics of 726 CB units before cryopreservation and after thawing

Table 1 shows the TNC and CD34+ cell counts, recovery rate of TNC and CD34+ cells, viability of TNC before cryopreservation and after thawing and viability of CD45+ and CD34+ cells after thawing of CB units.

**TABLE 1:** Characteristics of 726 cord blood units.

Variable	Mean ± SD	Median	Range
Cryopreservation period^a^ (days)	2303 ± 1245	2224	376–5772
Pre‐TNC^b^ (×10^8^/unit)	14.0 ± 4.1	13.4	5.4–33.6
Post‐TNC^c^ (×10^8^/unit)	13.7 ± 4.1	13.2	5.2–33.7
TNC recovery rate^d^ (%)	95.6 ± 5.2	97.4	70.1–100.0
Pre‐CD34+ cells^e^ (×10^6^/unit)	5.4 ± 4.1	4.5	0.2–37.7
Post‐CD34+ cells^f^ (×10^6^/unit)	4.6 ± 3.2	4.0	0.3–30.1
CD34+ cells recovery rate^d^ (%)	81.6 ± 18.1	83.8	11.5–100.0
Pre‐viability of TNC^g^ (%)	89.3 ± 2.5	89.0	80.0–95.0
Post‐viability of TNC^h^ (%)	86.3 ± 2.6	86.0	77.0–93.0
△Viability of TNC^i^ (%)	3.1 ± 2.5	3.0	0.0–12.0
Viability of CD45+ cells after thawing (%)	60.5 ± 12.6	61.0	24.1–92.6
Viability of CD34+ cells after thawing (%)	91.2 ± 7.5	93.0	15.9–100.0

Abbreviation: TNC, total nucleated cell.

^a^ From donation to pre‐transplantation examination.

^b^ TNC count before cryopreservation.

^c^ TNC count after thawing.

^d^ While TNC and CD34+ cells count recovery rate after thawing was sometimes greater than 100%, we chose to use an upper limit of recovery rate of 100%.

^e^ CD34+ cell count before cryopreservation.

^f^ CD34+ cell count after thawing.

^g^ Viability of TNC before cryopreservation by 0.4% trypan blue method.

^h^ Viability of TNC after thawing by 0.4% trypan blue method.

^i^ Difference in the viability of TNC by 0.4% trypan blue method before cryopreservation and after thawing.

The recovery rates of TNC and CD34+ cell counts were 95.6% ± 5.2% (range, 70.1%–100.0%) and 81.6% ± 18.1% (range, 11.5%–100.0%), respectively. According to the increase of the cryopreservation period, the recovery rate of TNC count did not change (*r* = 0.051, *p* = 0.168), whereas that of CD34+ cell count showed a positive correlation (*r* = 0.287, *p* <0.001). The mean viability of TNC before cryopreservation and after thawing for all the CB units were 89.3% and 86.3%, respectively. According to the increase of the cryopreservation period, the difference in the viability of TNC before cryopreservation and after thawing did not change (*r* = −0.072, *p* = 0.053). The viability of CD45+ and CD34+ cells was 60.5% ± 12.6% (range, 24.1%–92.6%) and 91.2% ± 7.5% (range, 15.9%–100.0%), respectively. The viability of CD45+ cells showed a positive correlation (*r* = 0.304, *p* <0.001), whereas the viability of CD34+ cells showed a negative correlation (*r* = −0.306, *p* < 0.001).

### Quality changes of CB units according to the cryopreservation period groups

Table 2 shows the recovery rates of TNC and CD34+ cells, as well as the difference in the viability of TNC before cryopreservation and after thawing according to the cryopreservation period group (4‐year intervals).

**TABLE 2:** Recovery rates of total nucleated cell and CD34+ cells and the difference in the viability of total nucleated cell before cryopreservation and after thawing of cord blood units according to the cryopreservation period group.

Cryopreservation period group	*n*	TNC recovery rate^a^ (%)	CD34+ cells recovery rate^a^ (%)	△Viability of TNC^b^
		Mean ± SD (range)	Mean ± SD (range)	Mean ± SD (range)
1–4 years	244	95.2 ± 5.5 (70.1–100.0)	75.0 ± 20.7 (11.5–100.0)	3.3 ± 2.7 (0.0–12.0)
5–8 years	240	96.0 ± 5.0 (79.1–100.0)	84.0 ± 16.3 (32.9–100.0)	3.1 ± 2.4 (0.0–12.0)
9–12 years	196	95.8 ± 4.8 (78.4–100.0)	82.8 ± 15.1 (38.6–100.0)	3.2 ± 2.4 (0.0–10.0)
13–16 years	46	95.5 ± 6.4 (70.4–100.0)	98.2 ± 5.7 (68.5–100.0)	2.4 ± 1.8 (0.0–6.0)
Total	726	95.6 ± 5.2 (70.1–100.0)	81.6 ± 18.1 (11.5–100.0)	3.1 ± 2.5 (0.0–12.0)

Abbreviation: TNC, total nucleated cell.

^a^ While TNC and CD34+ cells count recovery rate after thawing was sometimes greater than 100%, we chose to use an upper limit of recovery rate of 100%.

^b^ The difference in the viability of TNC by 0.4% trypan blue method before cryopreservation and after thawing.

According to one‐way ANOVA, the mean recovery rate of TNC and the difference in the viability of TNC before cryopreservation and after thawing of the CB units did not differ significantly among the cryopreservation period groups (*p* = 0.391 and 0.133, respectively). The mean recovery rate of CD34+ cells in the CB units was significantly different among the cryopreservation period groups (*p* < 0.001) according to one‐way ANOVA. A multiple comparison analysis showed significant differences in the mean recovery rate of CD34+ cells among the cryopreservation period groups (*p* < 0.001 for all comparisons), except for the comparison between the 5–8 year group and the 9–12 year group (*p* = 0.965). However, there was no negative trend in the mean recovery rate of CD34+ cells across the higher cryopreservation period groups.

Table 3 shows the viability of CD45+ and CD34+ cells after cryopreservation of CB units according to the cryopreservation period group.

**TABLE 3:** Change in the viability of CD45+ and CD34+ cells after thawing of cord blood units according to the cryopreservation period group.

Cryopreservation period group	*n*	Viability of CD45+ cells (%)	Viability of CD34+ cells (%)
		Mean ± SD (range)	Mean ± SD (range)
1–4 years	244	56.8 ± 11.0 (24.1–92.6)	93.3 ± 7.6 (15.9–100.0)
5–8 years	240	58.2 ± 11.8 (27.8–84.2)	91.8 ± 5.6 (74.6–100.0)
9–12 years	196	67.2 ± 12.8 (31.2–89.7)	89.2 ± 7.4 (60.0–99.2)
13–16 years	46	64.4 ± 12.0 (32.3–87.5)	86.1 ± 10.9 (49.4–97.7)
Total	726	60.5 ± 12.6 (24.1–92.6)	91.2 ± 7.5 (15.9–100.0)

**FIGURE 2:**
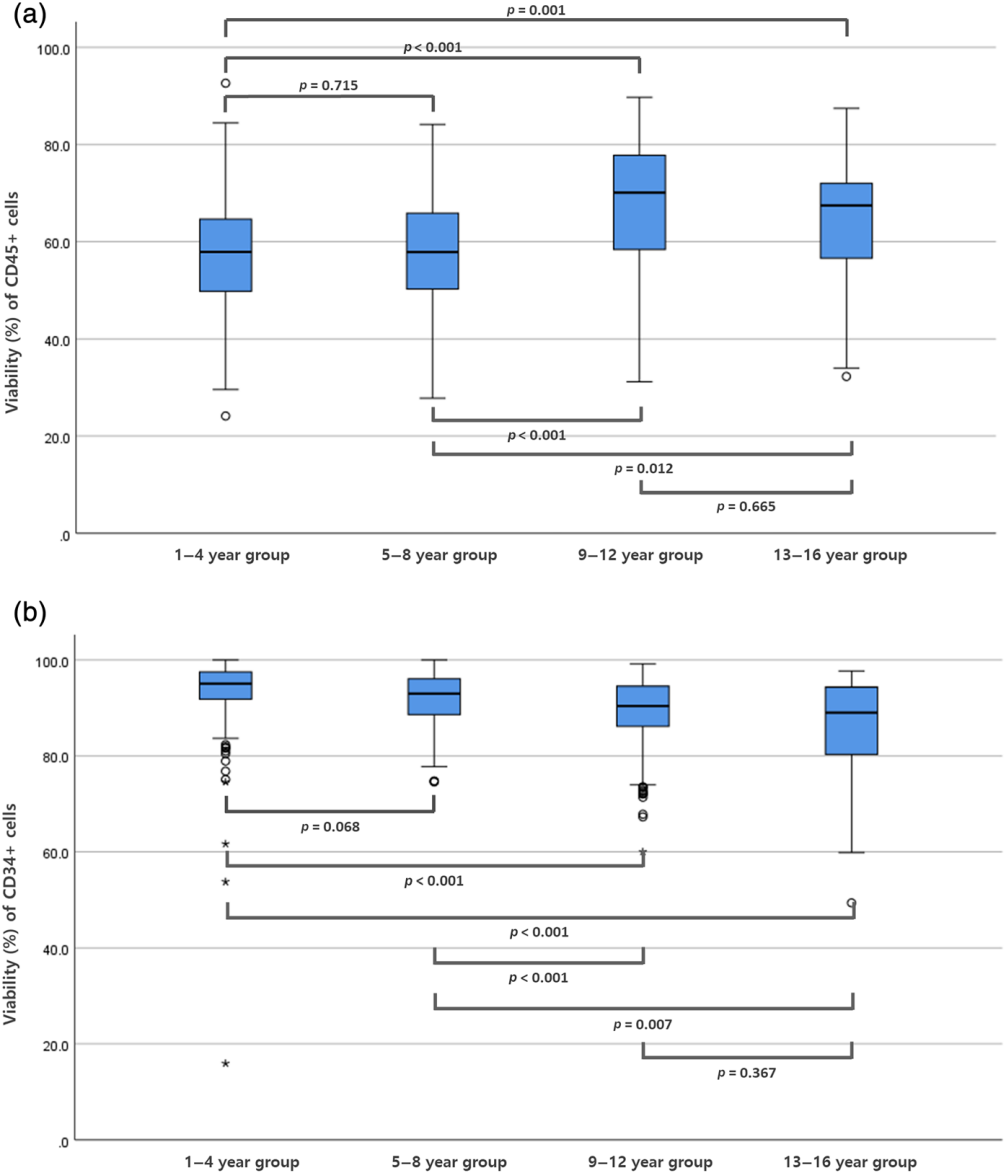
(a) Viability of CD45+ cells after cryopreservation of cord blood (CB) units according to the cryopreservation period group. Box and whisker graphs show median, 25th and 75th percentiles, range and outliers (circles) of the viability of CD45+ cells. (b) The viability of CD34+ cells after cryopreservation of CB units according to the cryopreservation period group. Box and whisker graphs show median, 25th and 75th percentiles, range and outliers (circles and asterisks) of the viability of CD34+ cells.

The mean viability of CD45+ cells in the CB units was significantly different among the cryopreservation period groups (*p* = 0.027) according to one‐way ANOVA. A multiple comparison analysis showed that the mean viability of CD45+ cells in the 1–4 year and 5–8 year groups was significantly lower than that in the 9–12 year and 13–16 year groups (Figure 2a). However, it is noteworthy that the mean viability of CD45+ cells did not exhibit a consistent decreasing or increasing trend across the cryopreservation period groups. In contrast, the mean viability of CD34+ cells showed a decreasing trend among the cryopreservation period groups. According to one‐way ANOVA, the mean viability of CD34+ cells in the CB units significantly differed among the cryopreservation period groups (*p* < 0.001). A multiple comparison analysis showed that the viability of CD34+ cells in the 1–4 year and 5–8 year groups was significantly higher than in the 9–12 year and 13–16 year groups (Figure 2b).

## DISCUSSION

We analysed whether there were the quality changes in the cryopreserved CB units during cryopreservation and investigated whether the cryopreservation period has an influence on the viability of CD45+ and CD34+ cells.

The studies at the New York Blood Center had indicated that there were no significant changes in the quality of the CB units that were cryopreserved for a maximum of 27 years as the haematopoietic stem cells [8, 9, 10, 11, 12]. Recently, they showed that the long‐term cryopreserved CB retained highly engraftable and functional haematopoietic stem cell potency through mouse models of transplantation [11]. The study at the José Carreras Cord Blood Bank indicated that there was no significant detrimental effect on engraftment after HSCT using the CB units that were cryopreserved for a maximum of 29.5 years [6]. Previously, we reported that there were no significant changes in the quality of CB units cryopreserved at our CB bank for up to 11.5 years of cryopreservation [13]. In this study, we could expand the period of no significant changes in the quality of cryopreserved CB units up to 16 years (5772 days).

The viability of CD45+ cells and CD34+ cells after thawing of CB units is used as a pre‐transplantation examination for cryopreserved CB units [5, 6, 14]. According to the NetCord‐FACT International Standards, assessment of CD34+ cell viability is required with a minimum viability threshold of 70% [5]. In accordance with the specifications of the PEI in Germany, assessment of both CD45+ cells and CD34+ cell viability is required with minimum viability criteria of 40% and 70%, respectively [6]. Similarly, the Kanto‐Koshinetsu Cord Blood Bank—one of the CB bands managed by the Japan Red Cross and the largest in Japan—has adopted the assessment of both CD45+ cells and CD34+ cell viability as a pre‐transplantation examination, following the approach of the PEI; however, their minimal acceptance criteria are more stringent, requiring at least 60% viability for CD45+ cells and at least 80% for CD34+ cells [15]. Several studies have demonstrated that post‐thaw CD34+ cell viability is a critical predictor of engraftment following CB transplantation [3, 4]. Units with CD34+ cell viability below 75% rarely contribute to engraftment, whereas those with higher viability are significantly more likely to engraft [3]. The mean post‐thaw viabilities of CD45+ cells (60.5%) and CD34+ cells (91.2%) observed in this study were well above the minimum acceptance criteria established by both the NetCord‐FACT International Standards and the PEI. These results demonstrate that the quality of the cryopreserved CB units meets or exceeds internationally recognized standards for transplantation.

The Kanto‐Koshinetsu Cord Blood Bank additionally assesses both TNC and CD34+ cell recovery rate, along with the CD45+ cells and CD34+ cell viability, as part of the pre‐transplantation examination [15]. Assessment of both TNC and CD34+ cell recovery rate is required with minimum criteria of 85% and 60%, respectively. The mean recovery rate of TNC (95.6%) and CD34+ cells (81.6%) observed in this study were well above the minimum acceptance criteria established by the Kanto‐Koshinetsu Cord Blood Bank. Because pre‐transplantation examination can only be performed using contiguous segment of each unit, which contains a small volume of highly concentrated CB, dilution of CB in the segment is required for pre‐transplantation examination. In addition, CB could be exposed to a different microenvironment during cryopreservation in the thin segment compared with in the main bag. These factors may lead to an underestimation of CD34+ cell count and consequently to an apparently low CD34+ cells recovery rate, rather than reflecting the association with the cryopreserved period. Therefore, we assume that the low recovery rate observed in some units is more likely due to variability at the individual level. It may be more appropriate to place greater emphasis on cell viability, rather than on recovery rate alone, as a pre‐transplantation quality parameter, which is in line with the requirement of the NetCord‐FACT and the PEI.

In this analysis, the cryopreservation period did not affect the proportion of viable CD45+ cells in the cryopreserved CB units. The proportion of viable cells among total CD34+ cells was higher than that among the total CD45+ cells. This finding is consistent with previous reports that haematopoietic progenitor cells, such as CD34+ cells, are more resistant to cryopreservation‐induced damage than differentiated cells like granulocytes, likely due to differences in membrane lipid and protein composition [16, 17]. Despite this relative resilience, the proportion of viable CD34+ cells in cryopreserved CB units tended to decrease with longer durations of cryopreservation. This trend of declining CD34+ cell viability with increasing cryopreservation period has been observed in previous studies as well [6, 18]. For instance, one investigation reported that CB units processed using current processing procedures showed a trend towards decreased CD34+ cell viability with increasing cryopreservation period, although this decline did not reach statistical significance [6]. Additionally, another study found that CB blood units cryopreserved for 10 years or more exhibit reduced CD34+ cell viability compared to those cryopreserved for shorter periods, although the differences between groups were not statistically significant [18]. Given these observations, it is critical to maximize the absolute number of CD34+ cells with the ISHAGE method at the time of cryopreservation. An increased starting cell dose can mitigate the impact of viability loss during long‐term storage, thus ensuring that an adequate number of CD34+ cells are available post‐thaw to support successful HSCT. In this context, even in Japan, where only CB units with a TNC count exceeding 14 × 10^8^ are cryopreserved, CD34+ cell counts are measured for units with a TNC count between 12 × 10^8^ and 14 × 10^8^, and those with a CD34+ cell count of at least 2.5 × 10^6^ are adopted as an additional cryopreservation criterion [15].

The functional capacity of CB units after thawing is commonly assessed by CFU assays, which evaluate the clonogenic and proliferative potential of haematopoietic stem and progenitor cells and have been shown to predict engraftment outcomes after CB transplantation [19, 20]. Several studies have demonstrated that higher post‐thaw CFU contents, particularly CFU‐granulocyte/macrophage (CFU‐GM), CFU‐granulocyte/erythrocyte/monocyte/megakaryocyte (CFU‐GEMM) and total CFU doses, are significantly associated with improved engraftment rates and faster haematopoietic recovery [9, 21]. In our analysis, partial correlation adjusted for cryopreservation period revealed significant associations between viable CD34+ cell proportion and total CFU (*r* = 0.109, *p* = 0.008) (data not shown). These results indicate that the proportion of viable CD34+ cells reflects CFU content and, consequently, serves as a surrogate marker for the haematopoietic potential of thawed CB units [5, 6, 14]. Therefore, determining the minimum pre‐cryopreservation CD34+ cell dose, considering anticipated viability losses over time, is critical for maintaining the desired quality of CB units for transplantation.

In conclusion, our study demonstrated that cryopreserved CB units at our bank cryopreserved for up to 16 years exhibit post‐thaw viabilities of CD45+ and CD34+ cells well above internationally recognized minimum thresholds, with CD34+ cell viability remaining higher than that of CD45+ cells. While CD45+ cell viability was unaffected by storage duration, CD34+ cell viability tended to decline over prolonged cryopreservation. To ensure long‐term quality, CB banks should secure CB units with sufficiently high CD34+ cell doses at the time of cryopreservation to offset potential viability loss during extended cryopreservation periods. Such measures will help secure the long‐term quality and clinical utility of CB units for HSCT.

## CONFLICT OF INTEREST STATEMENT

The authors declare no conflicts of interest.

## Data Availability

The data that support the findings of this study are available from the corresponding author upon reasonable request.
